# SynthRAD2025 Grand Challenge dataset: Generating synthetic CTs for radiotherapy from head to abdomen

**DOI:** 10.1002/mp.17981

**Published:** 2025-07-15

**Authors:** Adrian Thummerer, Erik van der Bijl, Arthur Jr Galapon, Florian Kamp, Mark Savenije, Christina Muijs, Shafak Aluwini, Roel J. H. M. Steenbakkers, Stephanie Beuel, Martijn PW Intven, Johannes A. Langendijk, Stefan Both, Stefanie Corradini, Viktor Rogowski, Maarten Terpstra, Niklas Wahl, Christopher Kurz, Guillaume Landry, Matteo Maspero

**Affiliations:** ^1^ Department of Radiation Oncology LMU University Hospital LMU Munich Munich Germany; ^2^ Department of Radiation Oncology Radboud University Medical Center Nijmegen The Netherlands; ^3^ Department of Radiation Oncology University Medical Center Groningen University of Groningen Groningen The Netherlands; ^4^ Department of Radiation Oncology and Cyberknife Center University Hospital of Cologne Cologne Germany; ^5^ Department of Radiotherapy University Medical Center Utrecht Utrecht The Netherlands; ^6^ Computational Imaging Group for MR Diagnostics & Therapy University Medical Center Utrecht Utrecht The Netherlands; ^7^ Radiation Physics, Department of Hematology, Oncology, and Radiation Physics Skåne University Hospital Lund Sweden; ^8^ Medical Radiation Physics, Department of Clinical Sciences Lund Lund University Lund Sweden; ^9^ Division of Medical Physics in Radiation Oncology Deutsches Krebsforschungszentrum (DKFZ) Heidelberg Germany; ^10^ Heidelberg Institute for Radiation Oncology (HIRO) and National Center for Radiation Research in Oncology (NCRO) Heidelberg Germany; ^11^ German Cancer Consortium (DKTK), Partner Site Munich a partnership between DKFZ and LMU University Hospital Munich Munich Germany; ^12^ Bavarian Cancer Research Center (BZKF) Munich Germany

**Keywords:** artificial intelligence, CBCT, CT, deep learning, image synthesis, MR

## Abstract

**Purpose:**

Medical imaging is crucial in modern radiotherapy, aiding diagnosis, treatment planning, and monitoring. The development of synthetic imaging techniques, particularly synthetic computed tomography (sCT), continues to attract interest in radiotherapy. The *SynthRAD2025* dataset and the accompanying SynthRAD2025 Grand Challenge aim to stimulate advancements in synthetic CT generation algorithms by providing a platform for comprehensive evaluation and benchmarking of synthetic CT generation algorithms based on cone‐beam CTs (CBCT) and magnetic resonance images (MRI).

**Acquisition and validation methods:**

The dataset comprises 2362 cases, including 890 MRI‐CT pairs and 1472 CBCT‐CT pairs of head‐and‐neck, thoracic, and abdominal cancer patients treated at five European university medical centers [UMC Groningen, UMC Utrecht, Radboud UMC (Netherlands), LMU University Hospital Munich, and University Hospital of Cologne (Germany)]. Images were acquired using a wide range of acquisition protocols and scanners. Pre‐processing, including rigid and deformable image registration methods, was performed to ensure high‐quality image datasets and alignment between modalities. Extensive quality assurance was performed to validate image consistency and usability.

**Data format and usage notes:**

All imaging data is provided using the MetaImage (.mha) file format, ensuring compatibility with common medical image processing tools. Metadata, including acquisition parameters and registration details, is available in structured comma‐separated value (CSV) files. To ensure dataset integrity, *SynthRAD2025* is split into training (65%), validation (10%), and test (25%) sets. The dataset is accessible through https://doi.org/10.5281/zenodo.14918088 under the *SynthRAD2025* collection.

**Potential applications:**

This dataset enables benchmarking and development of synthetic imaging techniques for radiotherapy applications. Potential use cases include sCT generation for MRI‐only and MR‐guided photon and proton radiotherapy, CBCT‐based dose calculations, and adaptive radiotherapy workflows. By incorporating data from diverse acquisition settings, *SynthRAD2025* supports the advancement of robust and generalizable image synthesis algorithms for clinical implementation, ultimately promoting personalized cancer care and improving adaptive radiotherapy workflows.

## 1 INTRODUCTION

Over the last decade, advancements in image‐guided and adaptive radiotherapy have significantly improved treatment outcomes for cancer patients, partially due to the introduction of image‐guided (daily) adaptive photon and proton radiotherapy.^1^ These approaches rely on accurate imaging to account for anatomical and physiological changes throughout treatment, enabling precise dose delivery to tumor volumes while sparing surrounding healthy tissues.^2^ Computed tomography (CT) imaging remains the gold standard for treatment planning, offering the electron density information critical for accurate dose calculations.^3^ However, frequent CT imaging is time‐consuming and costly, has an additional imaging dose burden for patients, and is usually unavailable directly on radiotherapy delivery machines.^4^

To address these challenges, alternative imaging modalities such as cone‐beam CT (CBCT) and magnetic resonance imaging (MRI) are increasingly used to replace CT acquisitions during treatment.^5^, ^6^ Compact CBCT systems can be easily integrated with treatment machines, providing volumetric patient images, and have become standard for daily pre‐treatment patient alignment.^7^, ^8^ However, CBCT image quality is usually inferior to diagnostic fan‐beam CT quality, mainly due to increased scatter and other CBCT imaging artifacts, which prevent the use of CBCT images for accurate dose calculations.^9^, ^10^ Recent advancements in clinically available CBCT hardware and software have enabled direct dose calculations in photon radiotherapy,^11^, ^12^ although this approach is not yet widely adopted.

MRI, on the other hand, offers superior soft‐tissue contrast and functional imaging capabilities without ionizing radiation. However, direct dose calculations on MRIs are impossible due to the lack of electron density information required for dose calculation algorithms.^13^ Still, there is an increasing interest in MR‐only radiotherapy workflows,^14^ and although more technically challenging to realize than compact CBCT systems, MR‐Linacs have proven that MRI can be efficiently combined with treatment machines and enable daily MR‐guided online adaptive photon radiotherapy.^15^ The combination of a treatment machine and an MRI is more challenging for proton therapy due to the interaction between magnetic fields and proton beams; however, research and development are ongoing, and MR‐guided proton therapy might become clinically available.^16^

The image quality limitations of CBCT and the absence of electron density information in MRI have sparked interest in generating so‐called synthetic CTs (sCT) from CBCT and MRI data to enable accurate dose calculations. Beyond generating electron density maps for dose calculations, sCTs have also proven valuable in facilitating organ‐at‐risk and target volume auto segmentation.^17^, ^18^ Numerous studies highlight artificial intelligence, particularly deep learning, as one of the most promising approaches for sCT generation.^5^ However, a lack of public datasets for CBCT and MR‐based sCT generation makes a fair and meaningful comparison of deep learning‐based sCT algorithms challenging. In 2023, the first edition of the *SynthRAD* challenge, *SynthRAD2023*, addressed this by providing the first large‐scale public multi‐center dataset to comprehensively compare sCT generation in brain and pelvic patients.^19^, ^20^ The *SynthRAD2025* challenge and dataset build upon the success of the *SynthRAD2023* challenge and provide a public dataset for three additional anatomical locations, head‐and‐neck, thorax, and abdomen, collected at five European university medical centers. The *SynthRAD2025* dataset aims to support and accelerate research in medical image synthesis for radiotherapy by providing high‐quality, curated, and paired CBCT‐to‐CT and MRI‐to‐CT datasets. The dataset facilitates the development, validation, and benchmarking of sCT generation algorithms, promoting advancements in radiotherapy and personalized cancer care.

## 2 ACQUISITION AND VALIDATION METHODS

### 2.1 Dataset overview

The *SynthRAD2025* dataset is part of the second edition of the SynthRAD deep learning challenge, which focuses on benchmarking MRI‐ and CBCT‐based sCT generation solutions (https://synthrad2025.grand‐challenge.org/). Similar to the previous *SynthRAD2023* challenge,^19^, ^20^ *SynthRAD2025* is structured into two tasks: Task 1 addresses MRI‐to‐CT conversion for MR‐only and MR‐guided photon and proton radiotherapy; Task 2 focuses on CBCT‐to‐CT translation for daily adaptive radiotherapy workflows. The *SynthRAD2025* challenge dataset provides data for sCT generation in head‐and‐neck, thoracic, and abdominal cancer patients. Imaging data was collected at radiation oncology departments of five European university medical centers, three from the Netherlands: UMC Groningen, UMC Utrecht, and Radboud UMC, and two from Germany: LMU University Hospital Munich and University Hospital of Cologne.

This study has been independently approved by all centers in accordance with the regulations of their respective institutional review boards or medical ethics committees.

The dataset comprises 2362 cases, where 890 are MRI‐CT pairs for task 1 and 1472 are CBCT‐CT pairs for task 2. The only inclusion criteria for the *SynthRAD2025* challenge datasets were treatment with some form of external beam radiotherapy (photon‐ or proton‐beam therapy) at one of the data‐providing centers and available imaging data from one of the respective anatomical regions. There were no further limitations on age, sex, or tumor characteristics, for example, type, size, location, and staging. Due to the large dataset size, we have collected a representative sample of patients treated at these radiation oncology departments. Datasets for head‐and‐neck, thorax, and abdomen subsets were mainly collected based on imaging protocols used in the respective region and institution. Due to this selection, patients from one region were occasionally imaged with the imaging protocol of other regions, for example, abdomen patients imaged with thorax protocol might be present in the thorax training dataset. However, only patients whose target volumes belonged to the respective anatomical region were selected for the test and validation sets.

**FIGURE 1:**
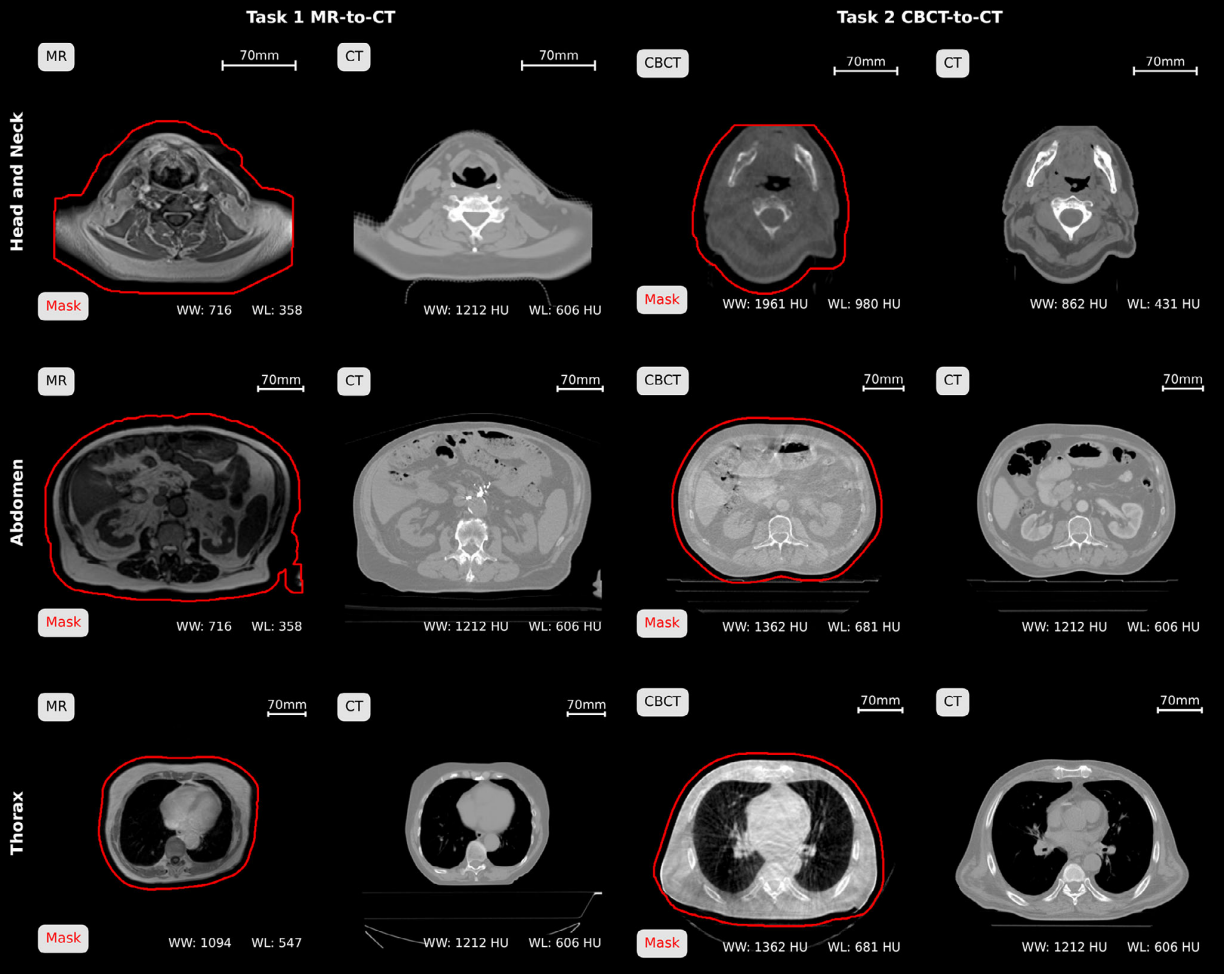
Example images for head‐and‐neck (top), abdomen (middle), and thorax (bottom) cases of Task 1 (left) and Task 2 (right) of the SynthRAD2025 dataset, with the contour of the provided patient outline mask in red.

In some centers, access to detailed patient characteristics was limited due to ethical considerations of data privacy and anonymization, preventing detailed statistics about age and sex distribution in the dataset. Figure 1 presents exemplary images for each task and anatomical region. For *SynthRAD2025*, the dataset was split into a training, validation, and test set, aiming at a split of 65/10/25%, respectively. However, this may slightly vary depending on data availability per center, task, and anatomy. To ensure the integrity of the *SynthRAD2025* challenge, initially, only the training dataset will be released publicly (details see Section 3.2). Table 1 presents the number of cases per set, task, anatomical region, and center. Data‐providing centers are abbreviated using the letters A to E. The assigned letter does not align with the order of centers mentioned above. A detailed description of dataset characteristics for each task and anatomy is provided in Sections 2.2 and 2.3.

**TABLE 1:** The number of cases collected at each center (letter from A to E) for training, validation, and test set for the three anatomical sites: Head‐and‐neck (HN), thorax (TH), and abdomen (AB).

Training																			
		HN						TH						AB					
Center		A	B	C	D	E	All	A	B	C	D	E	All	A	B	C	D	E	All
**Task**	**1**	91	0	65	65	0	**221**	91	91	0	0	0	**182**	65	91	19	0	0	**175**
	**2**	65	65	65	65	65	**325**	65	65	63	63	65	**321**	64	65	62	53	65	**309**

Validation																			
		HN						TH						AB					
Center		A	B	C	D	E	All	A	B	C	D	E	All	A	B	C	D	E	All
**Task**	**1**	14	0	10	10	0	**34**	14	14	0	0	0	**28**	10	14	3	0	0	**27**
	**2**	10	10	10	10	10	**50**	10	10	10	10	10	**50**	10	10	10	8	10	**48**

Testing																			
		HN						TH						AB					
Center		A	B	C	D	E	All	A	B	C	D	E	All	A	B	C	D	E	All
**Task**	**1**	35	0	25	25	0	**85**	35	35	0	0	0	**70**	25	35	8	0	0	**68**
	**2**	25	25	25	25	25	**125**	25	25	25	24	25	**124**	25	25	25	20	25	**120**

### 2.2 Task 1

Centers A, B, C, and D provided data for task 1, which comprises a variety of image scanners and acquisition protocols. MRIs from centers A, C, and D were acquired for treatment planning, mainly for defining target volumes. MRIs from Center B were acquired on a low‐field MR‐Linac capable of daily MR imaging and real‐time (2D) cine acquisition during treatment. MRIs were acquired with a T1‐weighted gradient echo or a balanced steady‐state free‐precession sequence and collected along with the corresponding planning CTs for all subjects. For centers A and D, the mean time difference between MRI and CT acquisitions was 10.4 and 7.8 days, respectively. This information was unavailable for center B due to data sharing regulations.

#### 2.2.1 Head‐and‐Neck (1HN)

In total, 340 MRI‐CT pairs from head‐and‐neck cancer patients were provided by centers A, C, and D. Image acquisition systems and parameters for MRIs and CTs are presented in Table 2. The data provided by Center C was acquired for diagnostic purposes with a small FOV and different immobilization devices, which makes this dataset specifically challenging for sCT generation. Center A and Center C used similar immobilization and table tops on MRI and CT scanners, providing a similar position on CT and MRI and improving the quality of the pre‐processed data. Center A provided 140 cases since no head‐and‐neck MRI was available from Center B.

**TABLE 2:** Imaging parameters for the head‐and‐neck MRIs and CTs in Task 1.

MRI—Head‐and‐Neck			
Parameter	Center A	Center C	Center D
Manufacturer	Philips	Siemens Healthineers	Siemens Healthineers
Model	Ingenia v5.4‐7	Avanto	Skyra (21), Prisma (79)
Field strength (T)	3	1.5/3	3
Sequence	T1w spoiled turbo gradient‐echo Dixon (TFE)	T1w turbo spin‐echo (TSE)	T1w radio‐frequency‐spoiled gradient echo Dixon (Vibe)
Acquisition	3D	2D	3D
Contrast	Yes	Yes	Yes
Flip angle (°)	10	150/160	9
Echo numbers	2	1	1
Echo time (ms)	1.4, 2.4	8.7/11	2.46
Repetition time (ms)	4.4–5.4	475–863	5.5
Inversion time IR (ms)	–	–	–
Number of averages	1	1‐3	1
Echo train length	2 (70), 60 (70)	2/3	2
Phase encoding steps	252 (70), 462 (70)	150–405	278
Bandwidth (Hz/px)	718–723	190–391	455
Voxel spacing (mm)	0.6–1.0 × 0.6–1.0 × 1.1–2.0	0.98‐1.17 × 0.98‐1.17 × 3.0	0.9 × 0.9 × 0.9–1.0
Acquisition matrix	252–462 × 252–462 × 190–250	256–384 × 192–288 × 67–320	256–264 × 256–264 × 256
Acquisition time (s)	83 (70), 287 (70)	82–202	–

CT			
Parameter	Center A	Center C	Center D
Manufacturer	Philips (100), Siemens (40)	Philips/Siemens	Siemens Healthineers
Model	Big Bore (100), Biograph40 (40)	Brilliance Big Bore Biograph40 Somatom Go.Open Pro	SOMATOM Definition AS (81), SOMATOM go.Open Pro (9)
kV	120	120	120
Tube current (mA)	128‐534	60‐496	76‐174
Exposure time (ms)	614–10 000	725–1475	1000–1250
CTDIvol (mGy)	15.1–27.5	3.8–42.6	–
Rows/Columns	512	512	512
Pixel spacing (mm, mm)	0.9–1.3 × 0.9–1.3	0.98–1.37 × 0.98–1.37	0.98 × 0.98
Slice thickness (mm)	2	3	2
Reconstruction diameter (mm)	451–700	500–700	500

The dataset is labeled with the prefix “1HN.” In parenthesis, the number of cases with a specific parameter is specified, along with the proprietary name of the sequence. A minus sign indicates unavailable or inapplicable parameters.

#### 2.2.2 Thorax (1TH)

Only two of the five data‐providing institutes had suitable thoracic MRIs. Two hundred eighty images were collected, with equal contributions from centers A and B. The respective image acquisition parameters are listed in Table 3.

**TABLE 3:** Imaging parameters for the thorax MRIs and CTs in Task 1.

MRI—Thorax		
Parameter	Center A	Center B
Manufacturer	Philips	ViewRay
Model	Ingenia v5.1‐7	MRidian
Field strength (T)	1.5	0.35
Sequence	T1w spoiled gradient‐echo Dixon (70, TFE)/ T1w radial fat‐suppressed gradient echo (70, VANE)	balanced steady‐state free‐precession sequence (bSSFP, TrueFISP)
Acquisition	3D	2D
Acquisition type	free‐breathing 3D	breath‐hold 3D
Contrast	No	No
Flip angle (°)	10‐12	60
Echo numbers	1	1
Echo time (ms)	2.3–4.7	1.27–1.62
Repetition time (ms)	5.5–7.4	3.0–3.8
Inversion time IR (ms)	–	–
Number of averages	1‐4	1
Echo train length	400 (70), 105–120 (70)	–
Phase encoding steps	320–460	175–232
Bandwidth (Hz/px)	718–723	385–604
Voxel spacing (mm)	0.9‐1.3 × 0.9 × 1.3 × 2.5‐3.0	1.5–1.63 × 1.5‐1.63 × 1.5‐3.0
Acquisition matrix	400–460 × 400–460 (70);280−300 × 280−300 (70)	200−310 × 234−360
		
Acquisition time (s)	188–340	17–25

CT		
Parameter	Center A	Center B
Manufacturer	Philips (136), Siemens (4)	Toshiba
Model	Big Bore (136), Biograph40 (4)	Aquilion/LB
Acquisition type	4D average or 20% phase (∼50%), breath‐hold 3D or free‐breathing (∼50%)	breath‐hold 3D
kV	120	120
Tube current (mA)	31–271	40–417
Exposure time (ms)	615–10 829	500–750
CTDIvol (mGy)	2.7–35.5	–
Rows/Columns	512	512
Pixel spacing (mm)	0.9–1.4 × 0.9–1.4	0.82−1.52 × 0.82–1.52
Slice thickness (mm)	2 (30) ‐ 3 (110)	3
Reconstruction diameter (mm)	461–700	500–700

The dataset is labeled with the prefix “1TH.” In parenthesis, the number of cases with a specific parameter is specified, along with the proprietary name of the sequence. A minus sign indicates unavailable or inapplicable parameters.

#### 2.2.3 Abdomen (1AB)

Centers A, B, and C provided 270 abdominal MRI‐CT pairs in total, while Center C could only provide 30 cases. Center A provided 95 cases, and Center B compensated for the low number of Center C cases, which were 140 MRI and CT acquisition parameters are described in Table 4.

**TABLE 4:** Imaging parameters for the abdominal MRIs and CTs in Task 1.

MRI—Abdomen			
Parameter	Center A	Center B	Center C
Manufacturer	Philips	ViewRay	Philips/Siemens
Model	Ingenia v5.1‐7	MRidian	Marlin(21);Avanto
Field strength (T)	1.5	0.35	1.5(21);1.5/3.0(9)
Sequence	T1w spoiled gradient‐echo Dixon (50, TFE)/ T1w radial fat‐suppressed gradient echo (50, VANE)	Balanced steady‐state free‐precession sequence (bSSFP, TrueFISP)	SE(21);SE/GR
Acquisition	3D	2D	3D(21);2D/3D
Acquisition type	free‐breathing 3D	breath‐hold 3D	free‐breathing 3D
Contrast	No (70), Yes (30)	No	No
Flip angle (°)	8‐12	60	90(21);49‐180
Echo numbers	1	1	1
Echo time (ms)	2.3–4.6	1.27–1.62	124(21);1.9–205
Repetition time (ms)	5.4–6.8	3.0–3.8	1300(21);480–2040
Inversion time IR (ms)	–	–	–
Number of averages	1–5	1	2(21);1–3
Echo train length	58–200	–	100(21);1‐34
Phase encoding steps	352–412	175–232	347(21)
Bandwidth (Hz/px)	433–725	385–604	820(21)
Voxel spacing (mm)	0.9–1.3 × 0.9–1.3 × 2.2–7.0	1.5–1.6 × 1.5‐1.6 × 3.0	0.64 × 0.64 × 2(21)
Acquisition matrix	336–412 × 336–412 × 130−273	200–310 × 234–360	347 × 347 × 110(21)
			
Acquisition time (s)	123–332	17–175	84–154(21)

CT			
Parameter	Center A	Center B	Center C
Manufacturer	Philips	Toshiba	Philips/Siemens
Model	Big Bore	Aquilion/LB	Brilliance Big Bore/Somatom Go.Open Pro
Acquisition type	4D average, midposition, or 20% phase	breath‐hold 3D	4D average (40%), free‐breathing 3D (60%)
kV	90–120	120	120
Tube current (mA)	47–305	40–420	68–429
Exposure time (ms)	614–10 091	500–750	421–11 912
CTDIvol (mGy)	15.1–79.6	–	5.6–141
Rows	512	512	512
Pixel spacing (mm)	0.9–1.4 × 0.9–1.4	0.6–1.4 × 0.6–1.4	0.98‐1.17 × 0.98–1.17
Slice thickness (mm)	2 (8) ‐ 3 (132)	3	2–3
Reconstruction diameter (mm)	500–700	320–700	500–700

The dataset is labeled with the prefix “1AB.” In parenthesis, the number of cases with a specific parameter is specified, along with the proprietary name of the sequence. A minus sign indicates unavailable or inapplicable parameters.

### 2.3 Task 2

Thanks to the widespread use of image‐guided radiotherapy based on CBCT in clinical practice, CBCTs were available in all five participating centers for all anatomical regions, leading to 1496 CBCT‐CT pairs. Data was acquired on three different treatment machines/CBCT systems, representing many clinically used CBCT scanners and acquisition protocols. Whenever available, the CBCT from the first treatment fraction was chosen to minimize the time between CT and CBCT imaging. For centers A, D, and E, mean time differences between CBCT and CT acquisition were 6.4, 11.0, and 0.3 days, respectively. This information was unavailable for centers B and C due to data sharing regulations.

#### 2.3.1 Head‐and‐Neck (2HN)

The Head‐and‐Neck CBCT subset features datasets from Elekta (center A, B, C, D) and Varian (center E) linear accelerators (linac) and an IBA proton therapy machine (center D). Table 5 lists the parameters of CBCT and CT image acquisition.

**TABLE 5:** Imaging parameters for the head‐and‐neck CBCTs and CTs in Task 2.

CBCT—Head‐and‐Neck					
Parameter	Center A	Center B	Center C	Center D	Center E
Manufacturer	Elekta	Elekta	Elekta	IBA (97), Elekta (3)	Varian
Model	XVI v5.x	XVI v5.52	XVI v5.x	Proteus P+, XVI v5.x	TrueBeam OBI
kVp	100–120	100	120	100	100–125
Tube current (mA)	12–20	10	10–20	160	11–20
Exposure time (ms)	10‐32	10	22	3225	7500–18 060
Rows/Columns	270	270	270	270‐512 x 270‐512	512 × 512
Pixel spacing (mm)	1 × 1	1 × 1	1 × 1	0.5‐1 × 0.5‐1	0.5‐0.9 x 0.5‐0.9
Slice thickness (mm)	1	1	1	2–2.5	2
Reconstruction diameter (mm)	270	270	N/A	260	262–465

CT‐					
Parameter	Center A	Center B	Center C	Center D	Center E
Manufacturer	Philips, Siemens	Toshiba	Philips, Siemens	Siemens Healthineers	TOSHIBA, Siemens
Model	Big Bore (90), Biograph40 (10)	Aquilion/LB	Brilliance Big Bore(93), Biograph40 (7)	SOMATOM Confidence(??) /Definition(??)	Aquilion/LB, Biograph128
kV	120	120	120	120	120
Tube current (mA)	55–531	40–300	56–444	19–219	25–200
Exposure time (ms)	615–1000	500–1000	922–1457	1000	500–1000
CTDIvol (mGy)	15.1–27.5	7.3–117.8	10.1–38.6	–	–
Rows/Columns	512	512	512	512	512
Pixel spacing (mm)	0.7‐1.4 × 0.7‐1.4	1.1‐1.4 × 1.1‐1.4	1‐1.2 x 1‐1.2	1‐1.6 x 1‐1.6	1‐1.5 x 1‐1.5
Slice thickness (mm)	2–3	1–3	2–3	2	3–5
Reconstruction diameter (mm)	444–700	550–700	350–700	500–800	531–780

The dataset is labeled with the prefix “2HN.” In parenthesis, the number of cases with a specific parameter. A minus sign indicates unavailable or inapplicable parameters.

#### 2.3.2 Thorax (2TH)

In the thoracic region, CBCTs were acquired with various treatment machines: Centers A, B, C, and D used an Elekta linac, Center D an IBA proton cyclotron, and Center E a Varian linac. Table 6 lists detailed image acquisition parameters.

**TABLE 6:** Imaging parameters for the thorax CBCTs and CTs in Task 2.

CBCT—Thorax					
Parameter	Center A	Center B	Center C	Center D	Center E
Manufacturer	Elekta	Elekta	Elekta	IBA (90), Elekta (7)	Varian
Model	XVI v5.x	XVI v5.x	XVI v5.x	Proteus P+, XVI v5.x	TrueBeam OBI
Acquisition type	free‐breathing 3D	free‐breathing 3D	free‐breathing 3D	average 4D (3), free‐breathing 3D	breath‐hold 3D (40%), free‐breathing 3D (60%)
kV	100–120	120	120	110–120	100–125
Tube current (mA)	20–40	40	10–40	16–320	13–80
Exposure time (ms)	10–40	40	16–40	10–5900	1710–18 120
Rows/Columns	270	410	135–410	270–768 × 270–768	512 × 512
Pixel spacing (mm)	1 × 1	1 × 1	1‐2 × 1‐2	0.46‐1 × 0.46‐1	0.5‐0.9 × 0.5‐0.9
Slice thickness (mm)	1	1	1‐2	2‐2.5	2
Reconstruction diameter (mm)	270	410	N/A	350–500	262–465

CT					
Parameter	Center A	Center B	Center C	Center D	Center E
Manufacturer	Philips (98), Siemens (2)	Toshiba	Philips/Siemens	Siemens	Toshiba, Siemens
Model	Big Bore (98), Biograph (2)	Aquilion/LB	Brilliance Big Bore(90)/Somatom Go.Open Pro(9)/Biograph40(1)	SOMATOM Confidence, Definition, go.Open Pro	Aquilion/LB, Biograph128
Acquisition type	breath‐hold 3D (25%) free‐breathing 3D (75%)	free‐breathing 3D	free‐breathing 3D	average 4D	breath‐hold 3D (40%), free‐breathing 3D (60%)
kV	100 (2)‐120 (98)	120	120	120	120
Tube current (mA)	35–295	40–440	33–502	21‐243	17–440
Exposure time (ms)	500–10 837	500–800	437–11 914	500–6222	500–1000
CTDIvol (mGy)	3.1–35.5	–	2.3–40.6	‐	‐
Rows/Columns	512	512	512/1024	512	512
Pixel spacing (mm)	0.8 × 1	1.0‐1.4 × 1.0‐1.4	0.5‐1.5 × 0.5‐1.5	0.8‐1.6 × 0.8‐1.6	1‐1.5 × 1‐1.5
Slice thickness (mm)	2(6) ‐ 3(94)	2.5‐3.0	2‐3	2	3–5
Reconstruction diameter (mm)	486–700	500–700	500–750	398‐800	500–780

The dataset is labeled with the prefix “2TH.” In parenthesis, the number of cases with a specific parameter. A minus sign indicates unavailable or inapplicable parameters.

#### 2.3.3 Abdomen (2AB)

The collected abdomen CBCTs were predominantly acquired on linear accelerators (Elekta and Varian), and only a minimal number of abdominal cancer patients were treated with proton therapy (IBA) in center D. Acquisition parameters of CBCTs and corresponding CTs are presented in Table 7.

**TABLE 7:** Imaging parameters for the abdomen CBCTs and CTs in Task 2.

CBCT—Abdomen					
Parameter	Center A	Center B	Center C	Center D	Center E
Manufacturer	Elekta	Elekta	Elekta	Elekta (70), IBA (11)	Varian
Model	XVI 5.x	XVI	XVI v5.x	XVI v5.x, Proteus P+	TrueBeam OBI
Acquisition type	free‐breathing 3D	free‐breathing 3D	free‐breathing 3D	4D average, free‐breathing 3D	breath‐hold 3D (25%), free‐breathing 3D (75%)
kV	100–120	120	120	120–125	125–140
Tube current (mA)	20–64	40	10–40	16‐320	15–99
Exposure time (ms)	10–40	40	20–40	10–5900	750‐18 280
Rows/Columns	270–410	410	270–410	270–768	512 × 512
Pixel spacing (mm)	1 × 1	1 × 1	1 × 1	0.65‐1 × 0.65‐1	0.5‐0.9 × 0.5‐0.9
Slice thickness (mm)	1	1	1	2‐2.5	2
Reconstruction diameter (mm)	270–410	410	N/A	270‐500	262–465

CT					
Parameter	Center A	Center B	Center C	Center D	Center E
Manufacturer	Philips (93), Siemens (7)	Toshiba, GE	Philips(93)/Siemens(7)	Siemens Healthineers, GE Medical	Toshiba
Model	Big Bore (93), Biograph (7)	Aquilion/LB, Discovery 690	Brilliance Big Bore Biograph40 (3) Somatom Go.Open Pro (4)	SOMATOM Confidence, Definition, go.Open Pro, Optima CT580	Aquilion/LB
Acquisition type	4D average, midposition or 20% phase	free‐breathing 3D	free‐ breathing 3D	4D average, free‐breathing 3D	breath‐hold 3D (25%), free‐breathing 3D (75%)
kV	90 (4), 100 (30), 120 (66)	120	120	80–140	120
Tube current (mA)	40–419	40–440	76–500	30–419	17–440
Exposure time (ms)	500–10 837	500–800	500–11 908	437–8720	500–1500
CTDIvol (mGy)	3.1–‐71	3.5–108.4	10–74.8	–	–
Rows/Columns	512	512	512	512	512
Pixel spacing (mm)	0.7 × 1.4	0.78 ‐ 1.37 × 0.78 ‐ 1.37	0.98‐1.47 × 0.98‐1.47x	0.77‐1.56 × 0.77‐1.56	1‐1.52 × 1‐1.52
Slice thickness (mm)	2(33) ‐ 3(67)	2‐3	3	2‐2.5	1‐5
Reconstruction diameter (mm)	452–700	381–‐700	500–700	395–800	530–780

The dataset is labeled with the prefix “2AB.” In parenthesis, the number of cases with a specific parameter. A minus sign indicates unavailable or inapplicable parameters.

### 2.4 Pre‐processing

The preprocessing workflow aimed to harmonize image parameters (e.g., voxel spacing, orientation), anonymize images, reduce file size, generate patient outlines, and prepare datasets for sCT evaluation. The preprocessing code is publicly available at https://github.com/SynthRAD2025/preprocessing.

Each participating center exported MRIs, CBCTs, and corresponding planning CTs from their clinical databases. For a subset of patients included in the testing phase of *SynthRAD2025*, radiotherapy treatment planning structures were exported and preprocessed. These structures are not included in the released dataset. Following the raw data export, the preprocessing pipeline involved the following key steps.

#### 2.4.1 Rigid registration

MRIs and CBCTs were rigidly registered to their corresponding planning CTs using the Elastix registration framework.^21^ Parameter files were tested and optimized for each task and anatomical region, and the final parameter files used in preprocessing are included in the public repository.

#### 2.4.2 Defacing

**FIGURE 2:**
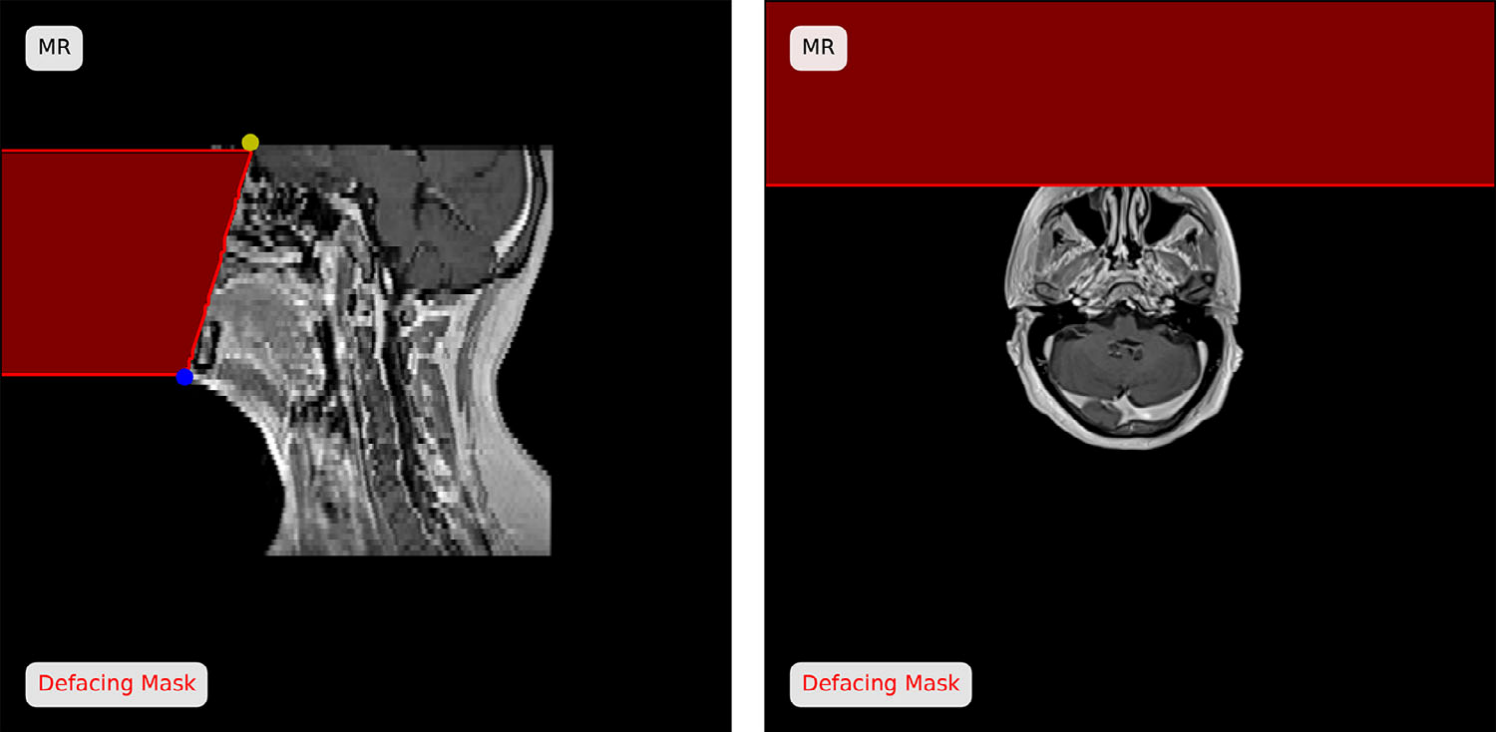
Example of the automatic defacing algorithm for the SynthRAD2025 dataset. The red area indicates the region overwritten with background values during the defacing. The yellow and blue markers indicate the points defining the defacing mask and were derived from the auto‐segmented brain (yellow) and mandible (blue) structures on the corresponding CT.

Images with visible facial structures were defaced to ensure patient anonymity using an automated algorithm developed for the *SynthRAD2025* dataset. This algorithm utilizes TotalSegmentator (version 2.3.0),^22^ a deep learning‐based CT auto‐segmentation model, to segment the skull and brain. Using these structures, the facial region was identified on the central sagittal slice of the brain mask by extracting the most anterior voxel of the brain (indicated by the yellow marker in Figure 2) and generating a bounding box around the skull mask. The anterior‐inferior corner of this bounding box was selected as the lower boundary of the face (see blue marker in Figure 2). The facial region was then defined as all voxels to the left of the line connecting these two points and was overwritten with the background intensity value (‐1024 for CT and 0 for MRI). The algorithm demonstrated strong robustness against patient positioning and orientation changes and did not require manual corrections. Figure 2 provides a visualization of the defacing process.

#### 2.4.3 Resampling

To standardize image resolution across the MRI, CBCT, and CT datasets, all images were resampled with a consistent voxel spacing of 1 × 1 × 3 mm.

#### 2.4.4 Outline segmentation

For each patient case, an outline mask was generated on the MR/CBCT image to define the volume used for evaluation and metric calculations during the validation and testing stage of the *SynthRAD2025* challenge. This mask was created automatically using histogram‐based thresholding, followed by morphological erosion and dilation operations. The threshold value varied between centers' anatomical regions and had to be manually tuned for some patients. The final mask was dilated further to include surrounding air, ensuring that sCT models accurately reconstruct the patient outline rather than relying on the mask itself. The automated process can result in minor inaccuracies and variable dilation margins for some patients. Given the large dataset size, manual corrections of masks were not feasible. The supplementary materials contain histograms of the volume of dilated patient masks (Figure S1), the volume of cropped images (Figure S2), average dilation margins (Figure S3), and examples of observed masking errors (Figure S4).

#### 2.4.5 Cropping

To minimize file and dataset size, patient images were cropped to a 10 pixels‐extended bounding box of the patient outline mask (described in Section 2.4.4).

#### 2.4.6 File conversion

Images were compressed and saved in the MetaImage format for the final datasets with the “.mha” extension. Pixel data was stored in INT16 to further reduce the file size.

#### 2.4.7 Deforming CT to MRI/CBCT

To evaluate image similarity and dose calculation accuracy, CTs were deformed to match the anatomy of the input MR or CBCT and generate ground truth CTs. This step reduced anatomical differences between synthetic and ground‐truth CTs. Deformable image registration was performed using the Elastix framework,^21^ and parameter files are publicly available in the source code. To avoid bias towards paired training approaches, deformed CTs are not provided for the training dataset and will only be released as part of the validation and testing dataset for *SynthRAD2025*.

### 2.5 Data validation

The *SynthRAD2025* dataset was designed to provide a representative sample of radiotherapy patients sourced from multiple international radiation oncology departments. Inclusion criteria were intentionally broad, including patients with even image artifacts or implants, provided the images were deemed suitable for sCT generation. The dataset was validated by focusing on image and preprocessing quality checks, with particular emphasis on the accuracy of the defacing algorithm to ensure patient anonymity and prevent re‐identification. Therefore, all datasets underwent visual checks by the respective institutions to ensure proper removal of facial features.

Further quality assurance involved generating overview images containing central axial, sagittal, and coronal slices from CBCT/MRI, CT, and the patient outline mask. These overviews also included overlaid CBCTs/MRIs and CTs to assess registration accuracy visually. It is important to note that these overviews are unsuitable for image intensity quantification due to inherent differences in intensity and contrast between imaging modalities. However, they are distributed as part of the *SynthRAD2025* dataset (Section 3.1). The large dataset volume limited the quality checks to three planes per image and patient.

Available image acquisition parameters were extracted from the original Dicom files and provided in .xlsx files for each dataset. The availability of organs‐at‐risk (OAR) and target structures and the accuracy of deformable image registration, which was visually assessed for all patients, guided the selection of patient cases for training, validation, and test sets.

During the visual control, the following observations were made: (1) In some cases, the position of the arms varied between MR/CBCT and CT acquisitions. (2) Image artifacts, such as those caused by metal implants, were present in a limited number of cases. (3) Depending on the definition of anatomical regions and imaging protocols in each center, some thoracic cases are included in the abdominal dataset and vice versa. (4) Variations among patients affected the automatic thresholding process for the definition of the body mask, resulting in the possible inclusion of couch structures or the exclusion of lung regions. As a result of the automatic thresholding and the varying thresholds used, the final dilation margins around the patient outline are different among patients and dataset. (5) The 1HN subset of center C included MRIs with a limited field of view, making rigid and deformable registration particularly challenging. These cases may be challenging for sCT generation. (6) Patient outline masks in subsets 1AB and 1TH of center B were cropped in the inferior‐superior direction due to varying MRI intensities and frequent artifacts at the edge of the FOV. In some cases, the cropped mask still includes artifacts, or the cropping might remove regular slices.

## 3 DATA FORMAT AND USAGE NOTES

### 3.1 Data structure and file formats

**FIGURE 3:**
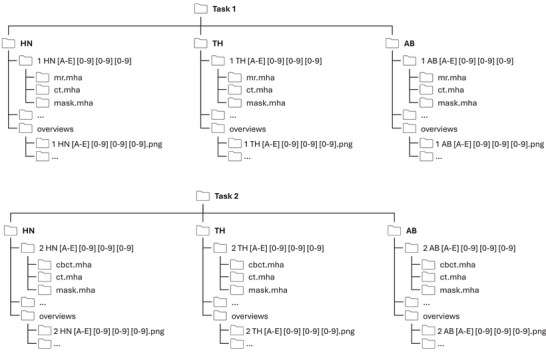
Folder structure of the SynthRAD2025 training dataset, split based on task and anatomy.

Figure 3 presents the directory structure of the *SynthRAD2025* training dataset. Similar to the *SynthRAD2023* challenge, the dataset is split into the two investigated tasks: Task 1 directory contains all MRI cases, and Task 2 contains all CBCT cases. Within each task, individual folders exist for each anatomical region: head‐and‐neck (HN), thorax (TH), and abdomen (AB). Within these anatomy directories, individual folders exist per case. Each case was assigned a unique seven‐letter alphanumeric code: a task identifier (1 or 2), a region identifier (HN, TH, or AB), a center identifier, and a three‐digit patient ID. Each patient folder contains the input image (mr.mha or cbct.mha), the corresponding CT (ct.mha), and the patient outline mask (mask.mha). The patient overview images and a spreadsheet with image acquisition parameters are provided in an overview directory in each region folder. The deformed CT (ct_def.mha) will also be included in the patient directories for the validation and testing datasets.

The dataset is provided under two different licenses. Data from centers A, B, C, and E is provided under a CC‐BY‐NC 4.0 International License (creativecommons.org/licenses /by‐nc/4.0/ https://creativecommons.org/licenses/by‐nc/4.0/). Table 8 provides an overview of the release dates and the files included in the SynthRAD2025 training, validation, and test set.

**TABLE 8:** Files, release dates, and links to the dataset download for training, validation, and test sets of centers A, B, C, and E.

Subset	Files	Release date	Link
Training	Input, CT, Mask	01.03.2025	https://doi.org/10.5281/zenodo.14918213
Validation	Input, Mask	01.06.2025	https://doi.org/10.5281/zenodo.14918504
Validation	CT, Deformed CT	01.03.2030	https://doi.org/10.5281/zenodo.14918605
Testing	Input, CT, Deformed CT, Mask	01.03.2030	https://doi.org/10.5281/zenodo.14918722

Data from center D is provided with a limited license that allows the use of the data solely for the challenge duration and is only valid as long as the challenge is active. Afterward, the data download will be deactivated, and the data must be deleted. After requesting participation in the challenge on the SynthRAD2025 website, participants can access the download link for center D at https://synthrad2025.grand‐challenge.org/data/.

### 3.2 Usage notes

All images are compressed files with .mha extension that can be read, written, and modified using the ITK open‐source framework (https://itk.org).^23^ For various programming languages, such as Python, R, Java, C++, etc., SimpleITK^24^ provides a simplified interface to ITK (https://simpleitk.org/). The pre‐processing scripts in the SynthRAD repository contain examples of basic image processing using SimpleITK. Several graphical user interface‐based applications exist for viewing .mha images, including 3DSlicer (https://www.slicer.org/), itksnap (http://www.itksnap.org/), and vv (https://github.com/open‐vv/vv).

## 4 DISCUSSION

The *SynthRAD2025* dataset is a comprehensive resource designed to advance research on sCT generation for radiotherapy. It includes detailed imaging data that supports developing, refining, and benchmarking algorithms for CT image synthesis. This initiative addresses a critical gap in the field by providing a large‐scale, multicenter, multi‐vendor, and publicly available dataset. It enables researchers to develop, validate, and benchmark sCT generation algorithms for MRI‐to‐CT and CBCT‐to‐CT tasks. Below, we discuss this work's implications, strengths, limitations, and future directions.

### 4.1 Implications for adaptive radiotherapy

The *SynthRAD2025* dataset has the potential to accelerate advancements in adaptive radiotherapy by addressing two key challenges: the limitations of CBCT image quality and the lack of electron density information in MRI. By facilitating the development of robust sCT generation algorithms, this dataset can improve the accuracy of dose calculations in MR‐guided and CBCT‐guided adaptive radiotherapy workflows. This is particularly relevant for proton therapy, where precise dose delivery is critical due to the sensitivity of proton beams to anatomical changes.^25^ Including multiple anatomical regions (head‐and‐neck, thorax, and abdomen) and data from various international institutions ensures that the dataset is representative of diverse clinical scenarios, making it a valuable resource to develop and benchmark robust algorithms for photon and proton radiotherapy.

### 4.2 Strengths of the dataset

The multi‐modal and multicenter *SynthRAD2025* dataset includes data from five European university medical centers, ensuring diversity in imaging protocols, patient populations, and treatment machines. This multicenter approach enhances the generalizability of algorithms developed using the dataset, potentially reducing the hurdle of translating research results into clinical practice. With almost 2400 cases, the *SynthRAD2025* dataset is the most extensive curated and publicly available dataset specifically targeted at sCT generation in radiotherapy. The rigorous automatic preprocessing pipeline followed by manual quality control ensures high‐quality and standardized data, including rigid registration, defacing, resampling, and outline segmentation. The separation into two separate tasks, addressing MRI‐to‐CT and CBCT‐to‐CT conversion, allows researchers to focus on specific challenges associated with each modality, such as the lack of electron density in MRI or the artifacts in CBCT, individually. Furthermore, it enables the investigation of which deep learning model suits each task best. The dataset is publicly available under open licenses, promoting transparency and reproducibility in research. The preprocessing code and parameter files are also publicly available, allowing the reproduction of the research and the reuse of private data with similar characteristics.

### 4.3 Limitations and challenges

While the multicenter design is a strength, it also introduces variability in imaging protocols, such as differences in MRI sequences, CBCT acquisition parameters, and CT reconstruction methods. Furthermore, even within the centers, imaging protocols and scanners often vary, limiting the number of datasets with homogenous image characteristics. This heterogeneity makes it challenging to develop universally applicable sCT generation algorithms.

Due to ethical considerations and data privacy concerns that vary among countries and centers, detailed patient characteristics, for example, age, sex, tumor type, and staging, are not uniformly available across the dataset. This limits the ability to perform subgroup analyses or evaluate algorithm performance in specific patient populations over the whole dataset. Whenever possible, patient characteristics were included in the metadata files.

Although the preprocessing pipeline was designed to harmonize the data, some steps, such as resampling, defacing, and outline segmentation, may partially deteriorate the data quality. For example, while robust, the automated defacing algorithm may occasionally remove non‐facial structures, and the patient outline masks may have variable dilation margins or include structures outside the patient, for example, the treatment couch.

Deformed CTs are not provided for the training dataset in the *SynthRAD2025* challenge to avoid bias toward paired deep learning training approaches. While this ensures a fair evaluation of sCT algorithms, it may limit the ability to train models that rely on deformable image registration and require extra steps from the dataset user to perform deformable registration and validate its results. The deformable image registration pipeline used for the validation and test set has been made publicly available to facilitate the participants.

### 4.4 Future directions

The *SynthRAD2025* dataset provides an excellent foundation for benchmarking existing and emerging sCT generation algorithms even beyond the *SynthRAD2025* Grand Challenge,^26^ as most parts of the data will stay publicly available. Future research should focus on integrating state‐of‐the‐art sCT generation algorithms into clinical workflows, particularly for online adaptive radiotherapy. This includes evaluating these algorithms' computational efficiency, robustness against outliers and artifacts, and clinical feasibility in real‐time treatment scenarios. While the series of SynthRAD datasets and accompanying challenges already covers five anatomical regions (brain, head‐and‐neck, thorax, abdomen, and pelvis), future iterations could further expand the datasets to include additional regions, such as extremities, special patient populations, for example, pediatric patients, or extend to other imaging modalities, such as ultrasound and PET. This would further enhance the dataset's applicability to a broader range of clinical scenarios.

## 5 CONCLUSION

The *SynthRAD2025* dataset is a resource for the radiotherapy research community. It offers a comprehensive and publicly available dataset for sCT generation. This dataset can drive advancements in personalized cancer care by addressing challenges in image synthesis for radiotherapy. Researchers and other dataset users must be mindful of the dataset's limitations and aim to develop robust, generalizable, and clinically feasible algorithms. The release of the validation and test sets after the challenge will further enable the community to validate and refine their approaches.

## CONFLICT OF INTEREST STATEMENT

The authors have nothing to disclose.

## Supporting information



Supporting Information

## References

[1] Glide‐Hurst CK , Lee P , Yock AD , et al. Adaptive radiation therapy (ART) strategies and technical considerations: a state of the ART review from NRG Oncology. Int J Radiat Oncol Biol Phys. 2021;109(4):1054‐1075. doi:10.1016/J.IJROBP.2020.10.021/ATTACHMENT/31BE5A6D-0DB1-4057-AEFB-D4872000AAAB/MMC2.PDF 33470210 PMC8290862

[2] Sonke JJ , Aznar M , Rasch C . Adaptive radiotherapy for anatomical changes. Semin Radiat Oncol. 2019;29(3):245‐257. doi:10.1016/J.SEMRADONC.2019.02.007 31027642

[3] Dobbs HJ , Parker RP , Hodson NJ , Hobday P , Husband JE . The use of CT in radiotherapy treatment planning. Radiother Oncol. 1983;1:141. 10.1016/s0167-8140(83)80016-4 6680218

[4] Zhou L , Bai S , Zhang Y , Ming X , Zhang Y , Deng J . Imaging dose, cancer risk and cost analysis in image‐guided radiotherapy of cancers. Sci Rep. 2018;8(1):1‐8. doi:10.1038/s41598-018-28431-9 29973695 PMC6031630

[5] Spadea MF , Maspero M , Zaffino P , Seco J . Deep learning based synthetic‐CT generation in radiotherapy and PET: a review. Med Phys. 2021;48(11):6537‐6566. doi:10.1002/MP.15150 34407209

[6] Keall PJ , Brighi C , Glide‐Hurst C , et al. Integrated MRI‐guided radiotherapy — opportunities and challenges. Nat Rev Clin Oncol. 2022;19(7):458‐470. doi:10.1038/s41571-022-00631-3 35440773

[7] Jaffray DA . Image‐guided radiotherapy: from current concept to future perspectives. Nat Rev Clin Oncol. 2012;9(12):688‐699. doi:10.1038/nrclinonc.2012.194 23165124

[8] Liu H , Schaal D , Curry H , et al. Review of cone beam computed tomography based online adaptive radiotherapy: current trend and future direction. Radiat Oncol. 2023;18(1):1‐12. doi:10.1186/S13014-023-02340-2/TABLES/2 37660057 PMC10475190

[9] Zhu L , Wang J , Xing L . Noise suppression in scatter correction for cone‐beam CT. Med Phys. 2009;36(3):741‐752. doi:10.1118/1.3063001 19378735 PMC2736744

[10] Zhu L , Xie Y , Wang J , Xing L . Scatter correction for cone‐beam CT in radiation therapy. Med Phys. 2009;36(6Part1):2258‐2268. doi:10.1118/1.3130047 19610315 PMC2832067

[11] Lustermans D , Fonseca GP , Taasti VT , et al. Image quality evaluation of a new high‐performance ring‐gantry cone‐beam computed tomography imager. Phys Med Biol. 2024;69(10):105018. doi:10.1088/1361-6560/AD3CB0 38593826

[12] Sijtsema ND , Penninkhof JJ , van de Schoot AJAJ , et al. Dose calculation accuracy of a new high‐performance ring‐gantry CBCT imaging system for prostate and lung cancer patients. Radiother Oncol. 2025;202:110596. doi:10.1016/j.radonc.2024.110596 39454887

[13] Bezin JV , Allodji RS , Mège JP , Schmidt MA , Payne GS . Radiotherapy planning using MRI. Phys Med Biol. 2015;60(22):R323. doi:10.1088/0031-9155/60/22/R323 26509844 PMC5137785

[14] Edmund JM , Nyholm T . A review of substitute CT generation for MRI‐only radiation therapy. Radiat Oncol. 2017;12(1):1‐15. doi:10.1186/S13014-016-0747-Y/FIGURES/6 28126030 PMC5270229

[15] Lagendijk JJW , Raaymakers BW , Van Den Berg CAT , Moerland MA , Philippens ME , Van Vulpen M . MR guidance in radiotherapy. Phys Med Biol. 2014;59(21):R349. doi:10.1088/0031-9155/59/21/R349 25322150

[16] Hoffmann A , Oborn B , Moteabbed M , et al. MR‐guided proton therapy: a review and a preview. Radiat Oncol. 2020;15(1):1‐13. doi:10.1186/S13014-020-01571-X/FIGURES/5 PMC7260752 32471500

[17] Dai X , Lei Y , Wynne J , et al. Synthetic CT‐aided multiorgan segmentation for CBCT‐guided adaptive pancreatic radiotherapy. Med Phys. 2021;48(11):7063‐7073. doi:10.1002/MP.15264 34609745 PMC8595847

[18] Hoffmans‐Holtzer N , Magallon‐Baro A , de Pree I , et al. Evaluating AI‐generated CBCT‐based synthetic CT images for target delineation in palliative treatments of pelvic bone metastasis at conventional C‐arm linacs. Radiother Oncol. 2024;192:110110. doi:10.1016/j.radonc.2024.110110 38272314

[19] Thummerer A , van der Bijl E , Galapon A , et al. SynthRAD2023 Grand Challenge dataset: generating synthetic CT for radiotherapy. Med Phys. 2023;50(7):4664‐4674. doi:10.1002/MP.16529 37283211

[20] Huijben EMC , Terpstra ML , Galapon AJ , et al. Generating synthetic computed tomography for radiotherapy: synthRAD2023 challenge report. Med Image Anal. 2024;97:103276. doi:10.1016/J.MEDIA.2024.103276 39068830

[21] Klein S , Staring M , Murphy K , Viergever MA , Pluim JPW . Elastix: a toolbox for intensity‐based medical image registration. IEEE Trans Med Imaging. 2010;29(1):196‐205. doi:10.1109/TMI.2009.2035616 19923044

[22] Wasserthal J , Breit HC , Meyer MT , et al. TotalSegmentator: robust segmentation of 104 anatomic structures in CT images. Radiol Artif Intell. 2023;5(5). doi:10.1148/RYAI.230024/ASSET/IMAGES/LARGE/RYAI.230024.FIG6.JPEG PMC10546353 37795137

[23] Yoo TS , Ackerman MJ , Lorensen WE , et al. Engineering and algorithm design for an image processing API: a technical report on ITK—the Insight Toolkit. Stud Health Technol Inform. 2002;85:586‐592. doi:10.3233/978-1-60750-929-5-586 15458157

[24] Lowekamp BC , Chen DT , Ibáñez L , Blezek D . The design of simpleITK. Front Neuroinform. 2013;7:73735. doi:10.3389/FNINF.2013.00045/BIBTEX PMC3874546 24416015

[25] Paganetti H , Botas P , Sharp GC , Winey B . Adaptive proton therapy. Phys Med Biol. 2021;66(22):22TR01. doi:10.1088/1361-6560/AC344F PMC8628198 34710858

[26] Thummerer A , Galapon AJ , Kurz C , et al. Synthesizing computed tomography for radiotherapy challenge (SynthRAD2025) International Conference on Medical Image Computing and Computer Assisted Intervention 2025 (MICCAI). 2024. doi:10.5281/ZENODO.14051075

