# Supplementary material for: SynthRAD2025 Grand Challenge dataset: Generating synthetic CTs for radiotherapy from head to abdomen
Source: Med Phys. 2025 Jul 15;52(7):e17981. doi: 10.1002/mp.17981 (PMC12264395; doi:10.1002/mp.17981)
Supplement: Supplementary file 1 — Supporting Information [file MP-52-0-s001.pdf]

Supplementary materials

Figure S1 presents volume histograms of the dilated patient outline masks for each task and anatomical region, calculated for the entire dataset, including training, validation and test sets. Noticeably, Head-and-Neck cases showed the smallest mask volumes, and task 1 showed larger average volumes than task 2. Figure S2 shows volume histograms of the cropped images also including data from all three sets (training, validation and testing). Similar trends as for the dilated mask volume can be observed in the cropped image volume, since the dilated mask was utilized to define the cropping region. Figure S3 presents histograms of the average surface distance (ASD) between dilated and undilated patient outline mask. Mean values for both tasks and all regions are as expected around 10 mm, with outliers due to variations in the thresholding levels or masking errors (see also Figure S4). Figure S4 presents some examples of errors and outliers in the patient outline segmentation process.

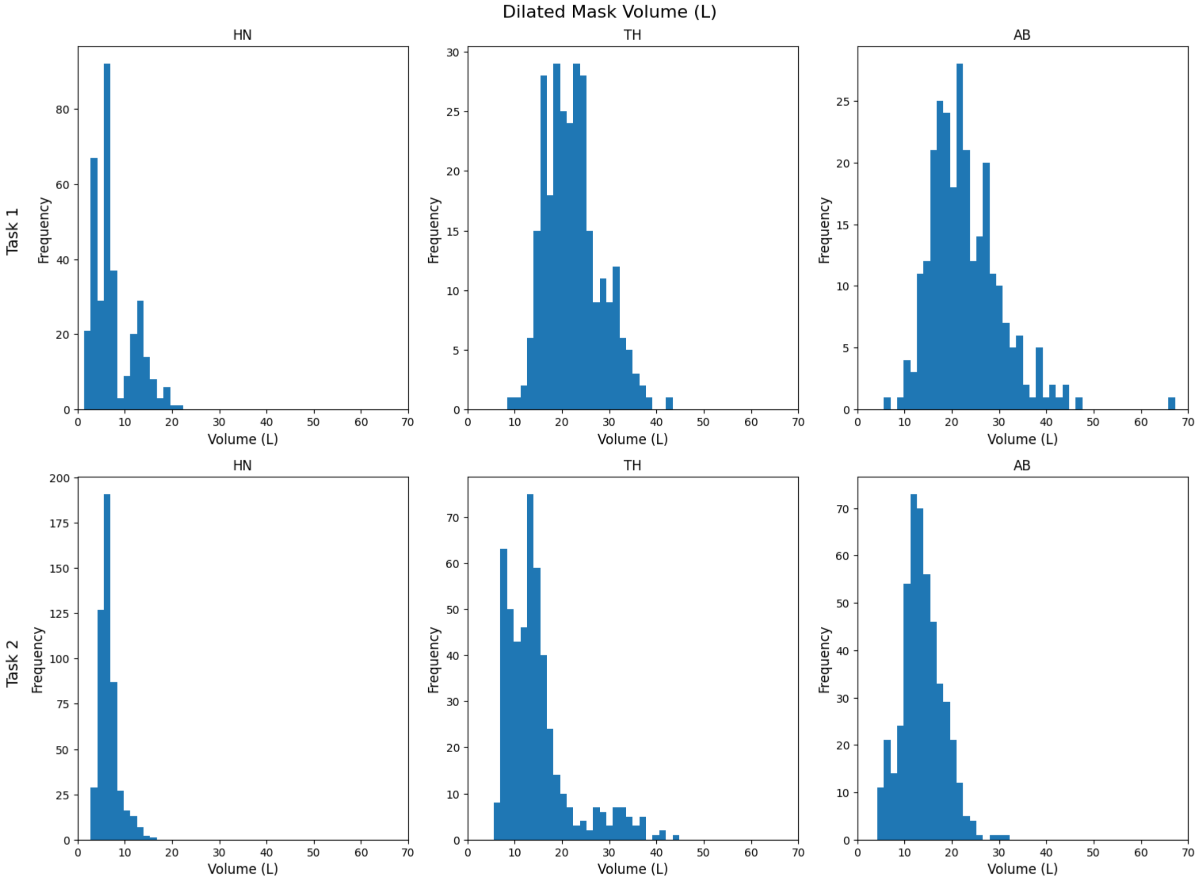

Figure S1: *Histograms of dilated mask volumes for each task and anatomy (HN = head and neck, TH = thorax, AB = abdomen).*

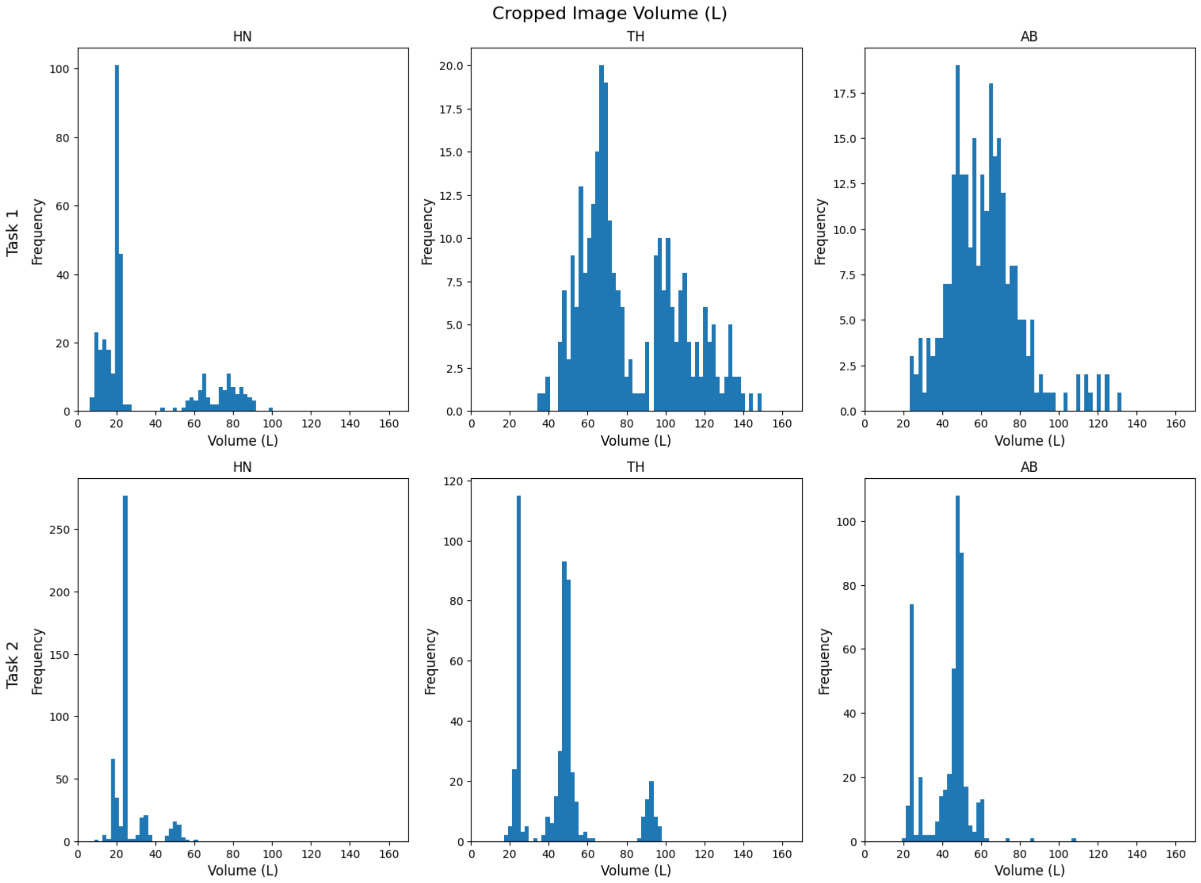

Figure S2: *Histograms of cropped image volumes for each task and anatomy (HN = head and neck, TH = thorax, AB = abdomen).*

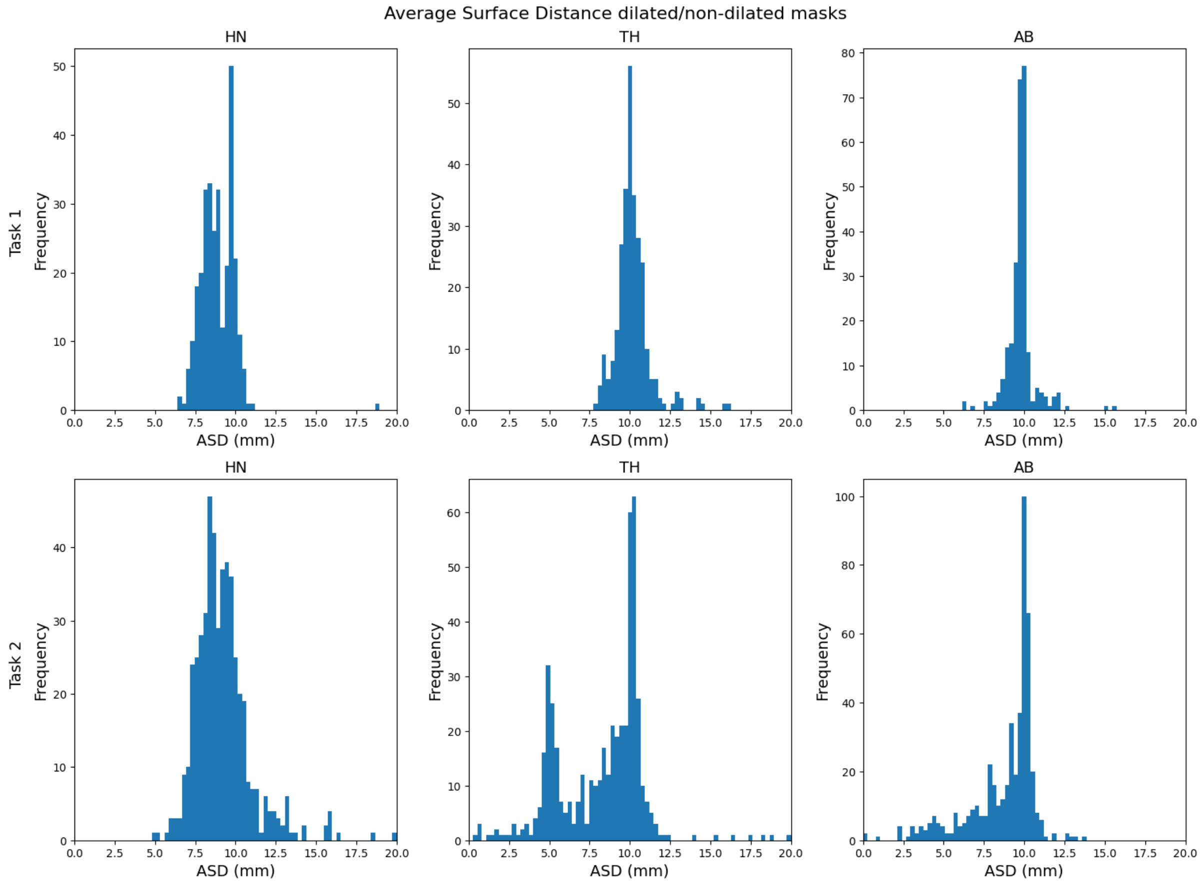

Figure S3: *Histograms of average dilation margins, calculated by computing the average surface distance (ASD) between dilated and non-dilated masks, for each task and anatomy (HN = head and neck, TH = thorax, AB = abdomen).*

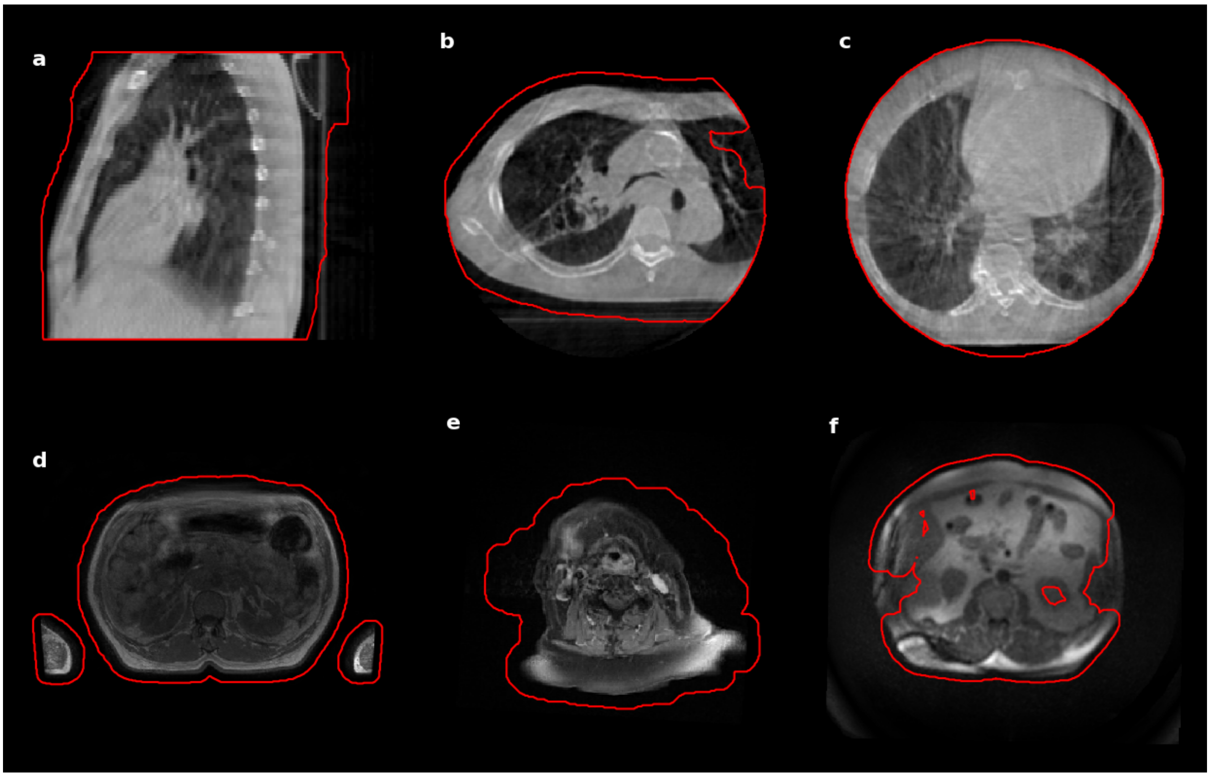

Figure S4: *Examples of observed masking and dilation errors:* ***(a)*** *the mask partially contains the patient couch/table,* ***(b)*** *in some cases with limited FOV the automatic masking missed parts of the lung if the lung was in contact with the FOV border,* ***(c)*** *in cases with very limited FOV no dilation could be performed,* ***(d)*** *in cases with varying arm positions between CT and MR/CBCT the arms might be included in the mask, but not in the corresponding CT or vice-versa,* ***(e)*** *large dilation margin due to noise/artifact in the images and* ***(f)*** *faulty mask in the most inferior slice due to image artifacts (e.g shadowing or streaks).*
